# Benefits of Prophylactic Cranial Irradiation in the MRI Era for Patients With Limited Stage Small Cell Lung Cancer

**DOI:** 10.3389/fonc.2022.833478

**Published:** 2022-02-28

**Authors:** Chao Qi, Wang Li, Hanming Li, Fengyun Wen, Lu Zhou, Xiaohu Sun, Hong Yu

**Affiliations:** ^1^School of Graduate, Dalian Medical University, Dalian, China; ^2^Department of Radiation Oncology, Cancer Hospital of China Medical University, Liaoning Cancer Hospital and Institute, Shenyang, China

**Keywords:** small cell lung cancer, prophylactic cranial irradiation, magnetic resonance imaging, brain metastases, patterns of failure

## Abstract

**Purpose:**

Previous studies have shown that prophylactic cranial irradiation (PCI) can improve the survival of patients with limited-stage small cell lung cancer (LS-SCLC). PCI is recommended for patients who respond well to chemoradiotherapy. However, whether PCI could be extrapolated to the LS-SCLC patients in the modern era of MRI is unknown. This study aimed to explore the value of PCI in patients with LS-SCLC in the era of brain MRI.

**Methods:**

This study included 306 patients with LS-SCLC at the Cancer Hospital of China Medical University. All patients received brain MRI at diagnosis and after radiochemotherapy to exclude brain metastases. A propensity score matching was performed to reduce the influence of potential confounders. Overall survival (OS), progression-free survival (PFS), and recurrence failure types were compared between PCI and non-PCI groups.

**Results:**

Among the 306 eligible patients, 81 underwent PCI, and 225 did not. After propensity score matching, there was no statistical difference in baseline data between the two groups, with 75 patients in each group. PCI did not achieve OS (median OS: 35 vs. 28 months, *p* = 0.128) or PFS (median PFS: 15 vs. 10 months, *p* = 0.186) benefits. During follow-up, 30 patients (20.0%) developed brain metastases, including 13 patients (17.3%) in the PCI group and 17 patients (22.7%) in the non-PCI group. Regarding death as a competitive risk, patients who received PCI had a lower cumulative incidence of brain metastasis than those who did not (3 years: 14.7% vs. 22.7%; Gray’s test, *p* = 0.007).

**Conclusions:**

When brain MRI was performed at diagnosis and pre-PCI, PCI could reduce the cumulative rate of brain metastases, but it did not achieve survival benefits for LS-SCLC patients.

## Introduction

Small cell lung cancer (SCLC) is a neuroendocrine tumor closely related to smoking, of which about 1/3 are limited-stage SCLC (LS-SCLC) at diagnosis. After initial treatment, SCLC patients have a high propensity for relapse and metastases. Dissemination to the brain is a preferential pattern of relapse and metastasizing for patients with SCLC, and 50%–60% of SCLC patients will have brain metastases within 2 years after diagnosis (1, 2).

A meta-analysis has shown that the application of prophylactic cranial irradiation (PCI) reduces the rate of brain metastases and significantly improves 3-year overall survival (OS) (15.3% vs. 20.7%) (3). Since then, PCI has been a category 1 recommendation for LS-SCLC patients exhibiting at least a partial response to initial radiotherapy and/or chemotherapy, according to the American Society for Radiation Oncology guidelines and National Comprehensive Cancer Network (4). However, in the above meta-analysis, the elimination of brain metastases was mainly based on brain CT or even brain X-rays at the first diagnosis, and they have not consistently used pretreatment MRI. Theoretically, this population may include patients with occult brain metastases. Whether PCI remains beneficial in the modern era of MRI is a point of contention (5–7). Recently, a retrospective study showed no survival benefit from PCI in patients who underwent brain MRI and/or CT to exclude brain metastases (7). Furthermore, PCI benefits are reduced if LS-SCLC patients are monitored by MRI and treated with stereotactic irradiation (SRI) (6). Therefore, whether PCI could be extrapolated to the LS-SCLC patients in the modern era of MRI is unknown. To address this question, we retrospectively analyzed the clinical data of 306 patients with LS-SCLC without brain metastasis diagnosed by brain MRI after initial chemoradiotherapy and then explored the value of PCI in LS-SCLC patients in the era of brain MRI to provide a reference for future clinical treatment.

## Materials and Methods

### Patients

We retrospectively reviewed LS-SCLC patients treated within our institution between January 2012 and January 2018. The inclusion criteria were as follows: pathologically diagnosed as SCLC; brain MRI was performed at diagnosis and after radiochemotherapy to exclude brain metastases; had any thoracic response (complete, partial response, or stable disease) to concurrent/sequential chemoradiotherapy. The exclusion criteria were as follows: combining with other malignant tumors, chemotherapy cycles ≤2, disease progression during chemoradiotherapy, and incomplete follow-up information. In brain MRI monitoring of intracranial status, regardless of whether PCI is performed, all patients were monitored by brain MRI every 3 months for 2 years, every 6 months for 3 years, and annually after 4 years. The study was approved by the Institutional Review Board of Cancer Hospital of China Medical University.

### Treatment

#### Chemotherapy

The chemotherapy regimen was mainly platinum-based chemotherapy and etoposide (cisplatin, 75 m^2^ d1 or 25 mg/m^2^ d1–3 + etoposide, 100 mg/m^2^ d1–3; carboplatin, area under the curve (AUC) = 5–6 d1 + etoposide, 100 mg/m^2^ d1–3), with chemotherapy cycles ≥3.

#### Thoracic Radiotherapy

Thoracic radiotherapy was delivered by 3D conformal or intensity-modulated radiotherapy (IMRT). In the supine position, a spiral CT (Philips Brilliance Big Bore CT, Eindhoven, Netherlands) simulation scan was performed from the cricothyroid membrane through the second lumbar vertebra with 3–5 mm of slice thickness. The obtained scan images have been uploaded to the Pinnacle workstation. The delineation of the target area refers to the imaging data before and after chemotherapy: gross tumor volume (GTV) included clinical and imaging visible tumor lesions (primary lesions and involved lymph nodes) based on contrast-enhanced CT scan; clinical target volume (CTV) was the 5-mm expansion of GTV and the complete involved lymphatic zone, which should be adjusted appropriately according to the adjacent anatomical structure. CTV is expanded 5 mm in a 3D direction to form planning target volume (PTV). Thoracic radiotherapy was given as 50–66 Gy in 25–33 daily fractions, 5 fractions/week. When thoracic radiotherapy reaches 4–5 weeks, lung CT should be re-examined to compare the changes in the lesions before and after radiotherapy. If the tumor shrinks significantly, repositioning, delineating the target area, and performing reduced field irradiation based on the patient’s condition.

#### Prophylactic Cranial Irradiation

PCI was recommended for LS-SCLC patients exhibiting any thoracic response (complete, partial response, or stable disease) to concurrent/sequential chemoradiotherapy within 1 month. CTV for PCI was the whole brain, and CTV expanded 3 mm was PTV. PCI was given as 25 Gy in 10 daily fractions (equivalent dose in 2-Gy fractions with an α/β ratio of 10 [EQD210] = 26 Gy) through the whole brain opposed lateral fields using 6–10 MV. The dose of radiosensitive organs at risk (OARs) was evaluated.

### Statistical Analysis

The clinical characteristics of the categorical variables were tested by the chi-square test or Fisher’s exact test for independence, and Student’s t-test tested the continuous variables. A propensity score match analysis was performed for patients with/without PCI. The Kaplan–Meier method was used to estimate the median OS (mOS) and progression-free survival (PFS), and the log-rank test was used for comparison between groups. The Cox proportional hazards regression model estimated the significant covariates. Brain failure risk (BFR) was modeled by the Fine and Gray subdistribution hazard method; deaths from any cause were considered a competing event. All tests were two-sided, and *p* < 0.05 was considered statistically significant. R statistical software version 4.1.0 (R Project for Statistical Computing) and SPSS 17.0 software (SPSS Inc., Chicago, IL, USA) were used.

## Results

### Patient Demographics and Treatment Characteristics

After exclusions, 306 patients were enrolled in our analysis. There were 206 (67.3%) male and 100 (33.3%) female patients. Of 306, 233 (76.1%) patients were clinical stage III before initial treatment. Treatment decisions were based on the results of the second cranial MRI, and finally, 81 patients received PCI (**Table 1**). The median follow-up time was 25 months (range: 4–102 months).

**Table 1:** Descriptive characteristics before and after propensity score matching.

Characteristic	Participants, No. (%)^a^					
	Before propensity score matching			After propensity score matching		
	PCI (n = 81)	non-PCI (n = 225)	*p*-Value	PCI (n = 75)	non-PCI (n = 75)	*p*-Value
Age						
<65 years	68 (84.0)	188 (83.6)	0.934	63 (84.0)	64 (85.3)	0.821
≥65 years	13 (16.0)	37 (16.4)		12 (16.0)	11 (14.7)	
Sex						
Female	26 (32.1)	74 (32.9)	0.897	22 (29.3)	16 (21.3)	0.260
Male	55 (67.9)	151 (67.1)		53 (70.7)	59 (78.7)	
Smoking index						
<400	42 (51.9)	126 (56.0)	0.520	37 (49.3)	30 (40.0)	0.250
≥400	39 (48.1)	99 (44.0)		38 (50.7)	45 (60.0)	
KPS						
<80	3 (3.7)	14 (6.2)	0.296	3 (4.0)	2 (2.7)	0.500
≥80	78 (96.3)	211 (93.8)		72 (96.0)	73 (97.3)	
Clinical stage						
I/II	27 (33.3)	46 (20.4)	**0.015**	23 (30.7)	25 (33.3)	0.726
III	54 (66.7)	179 (79.6)		52 (69.3)	50 (66.7)	
Obstructive pneumonia						
Yes	23 (28.4)	73 (32.4)	0.501	22 (29.3)	24 (32.0)	0.723
No	58 (71.6)	152 (67.6)		53 (70.7)	51 (68.0)	
Initial treatment						
Concurrent CRT	38 (46.9)	52 (23.1)	**0.001**	35 (46.7)	29 (38.7)	0.322
Sequential CRT	43 (53.1)	173 (76.9)		40 (53.3)	46 (61.3)	
Response to initial treatment						
CR	19 (23.5)	19 (8.4)	**0.001**	16 (21.3)	8 (10.7)	0.075
PR/SD	62 (76.5)	206 (91.6)		59 (78.7)	67 (89.3)	

PCI, prophylactic cranial irradiation; KPS, Karnofsky Performance Status; CRT, chemoradiotherapy; CR, complete response; PR, partial response; SD, stable disease.

The bold values inidicate p-value is less than 0.05.

In the non-PCI group, patients with clinical stage III (79.6% vs. 66.7%, *p* = 0.015), patients receiving sequential chemoradiotherapy (76.9% vs. 53.1%, *p* = 0.001), and patients with PR or SD efficacy (91.6% vs. 76.5%, *p* = 0.001) were all higher than those in the PCI group. After propensity score matching, there was no significant difference in baseline data between the two groups of 75 patients per group (**Table 1**).

### Survival Outcomes

**Figure 1:**
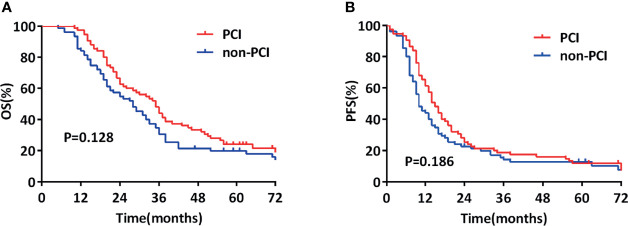
Comparison of overall survival (OS) **(A)** and progression-free survival (PFS) **(B)** of propensity-matched patients with limited-stage small cell lung cancer who did and did not undergo prophylactic cranial irradiation (PCI).

In the propensity-matched cohort (150), the mOS from LS-SCLC diagnosis was 31 months (95% CI, 26.1–35.9), with 3- and 5-year survival rates of 37.3% and 21.9%, respectively. The mOS was 35 and 28 months in the two groups (*p* = 0.128, **Figure 1A**), 3-year and 5-year survival rates were 44.0% and 24.0%, respectively, for those who received PCI and 30.7% and 19.9%, respectively, for those who did not receive PCI.

In the propensity-matched cohort (150), the median PFS (mPFS) from LS-SCLC diagnosis was 13 months (95% CI, 11.0–15.0), with 3- and 5-year survival rates of 16.5% and 12.3%, respectively. The mPFS was 15 and 10 months in the two groups (*p* = 0.186, **Figure 1B**), and the 3-year and 5-year survival rates were 18.7% and 11.9%, respectively, for those who received PCI 14.2% and 12.8%, respectively, for those who did not receive.

### Patterns of Failure

During the observation period, disease progression occurred in 103 (68.7%) patients, of which 52 (69.3%) patients and 51 (68.0%) patients were in the PCI group and non-PCI group, respectively. Patients with intracranial progression only, extracranial progression only, and intracranial combined with extracranial progression were respectively 4 (5.3%) cases, 39 (52.0%) cases, and 9 (12.0%) cases in the PCI group and 6 (8.0%) cases, 34 (45.3%) cases, and 11 (14.7%) cases in the non-PCI group (**Table 2**).

**Table 2:** Pattern of progression, after propensity score matching.

Pattern of progression	Total [n (%)]	PCI [n (%)]	Non-PCI [n (%)]
Only intracranial	10 (6.7)	4 (5.3)	6 (8.0)
Only extracranial	73 (48.7)	39 (52.0)	34 (45.3)
Intracranial and extracranial	20 (13.3)	9 (12.0)	11 (14.7)

PCI, prophylactic cranial irradiation.

**Figure 2:**
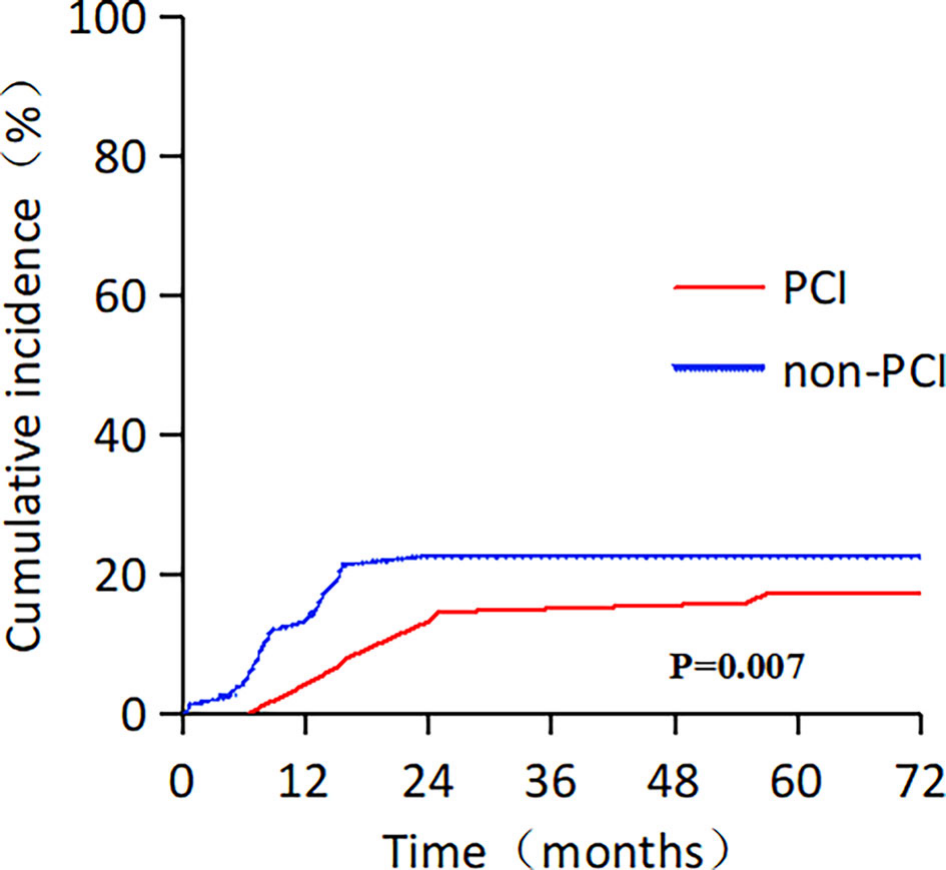
Cumulative incidence rate of distant brain metastasis, with death as competing risk, among patients with limited-stage small cell lung cancer.

Moreover, 30 patients (20.0 %) developed brain metastasis: 13 in the PCI group (17.3 %) and 17 in the non-PCI group (22.7 %). The median time for the central nervous system (CNS) failure was 18 and 9 months in the two groups (*p* = 0.001). Regarding death as a competitive risk, patients who received PCI had a lower cumulative incidence of brain metastasis than those who did not (3 years: 14.7% vs. 22.7%; Gray’s test, *p* = 0.007; **Figure 2**). The information on initial salvage treatments for brain metastases was as follows: 3 patients received brain radiotherapy, 1 patient received surgical in the PCI group, and 12 patients received brain radiotherapy in the non-PCI group.

### Prognostic Analysis

Univariate analysis showed that age (*p* = 0.037), TNM staging (*p* < 0.001), and efficacy of initial chemoradiotherapy (*p* = 0.020) were remarkable prognostic indicators associated with OS. Multivariate Cox regression analysis demonstrated that TNM staging (HR = 1.997, 95% CI: 1.340–2.976, *p* = 0.001) was independently associated with OS. Additionally, age (*p* = 0.032), TNM staging (*p* = 0.001), and efficacy of initial chemoradiotherapy (*p* = 0.025) were also significant prognostic indicators associated with PFS in the univariate analysis. Multivariate Cox regression analysis demonstrated that age (HR = 1.578, 95% CI: 1.000–2.490, *p* = 0.050) and TNM staging (HR = 1.806, 95% CI: 1.214–2.686, *p* = 0.004) were independently associated with PFS. However, PCI had nothing to do with the improvement of OS (HR = 0.760, 95% CI: 0.533–1.083, *p* = 0.129; **Table 3**) and PFS (HR = 0.815, 95% CI: 0.579–1.147, *p* = 0.241; **Table 4**).

**Table 3:** Univariate and multivariate analyses of factors influencing OS, after propensity score matching.

Variables	Univariate analysis		Multivariate analysis	
	χ^2^	*p*-Value	HR (95% CI)	*p*-Value
Age				
<65 or ≥65 years	4.367	**0.037**	1.576 (0.979–2.536)	0.061
Sex				
Female or Male	0.002	0.966		
Smoking index				
<400 or ≥400	0.413	0.520		
KPS				
<80 or ≥80	0.199	0.656		
Clinical stage				
I/II or III	13.210	<**0.001**	1.997 (1.340–2.976)	**0.001**
Obstructive pneumonia				
Yes or no	0.784	0.376		
Initial treatment				
Concurrent CRT or sequential CRT	0.076	0.783		
Response to initial treatment				
CR or PR/SD	5.391	**0.020**	1.546 (0.916–2.608)	0.103
PCI				
Yes or no	2.319	0.128	0.760 (0.533–1.083)	0.129

OS, overall survival; HR, hazard ratio; CI, confidence interval; PCI, prophylactic cranial irradiation; KPS, Karnofsky Performance Status; CRT, chemoradiotherapy; CR, complete response; PR, partial response; SD, stable disease.

The bold values inidicate p-value is less than 0.05.

**Table 4:** Univariate and multivariate analyses of factors influencing PFS, after propensity score matching.

Variables	Univariate analysis		Multivariate analysis	
	χ^2^	*p*-Value	HR (95% CI)	*p*-Value
Age				
<65 or ≥65 years	4.579	**0.032**	1.578 (1.000–2.490)	**0.050**
Sex				
Female or Male	0.122	0.727		
Smoking index				
<400 or ≥400	0.904	0.342		
KPS				
<80 or ≥80	0.002	0.960		
Clinical stage				
I/II or III	11.716	**0.001**	1.806 (1.214–2.686)	**0.004**
Obstructive pneumonia				
Yes or no	2.639	0.104		
Initial treatment				
Concurrent CRT or Sequential CRT	1.281	0.258		
Response to initial treatment				
CR or PR/SD	5.018	**0.025**	1.494 (0.900–2.479)	0.120
PCI				
Yes or no	1.752	0.186	0.815 (0.579–1.147)	0.241

OS, overall survival; HR, hazard ratio; CI, confidence interval; PCI, prophylactic cranial irradiation; KPS, Karnofsky Performance Status; CRT, chemoradiotherapy; CR, complete response;PR, partial response; SD, stable disease.

The bold values inidicate p-value is less than 0.05.

## Discussion

SCLC patients with brain metastases have a poor prognosis, and the mOS of patients receiving systemic treatment is only 8.5 months (8). A meta-analysis has shown that the application of PCI can reduce the cumulative brain metastasis rate and significantly improve OS and PFS (3). However, many patients lack brain imaging before PCI or only CT imaging. Furthermore, a single-center study performed pre-PCI brain MRI examinations on 40 consecutive LS-SCLC patients with CR to chemoradiotherapy and showed that 13/40 (32.5%; 95% CI: 18%–47%) patients had a relapse with brain metastases (9). Therefore, some patients in the meta-analysis may have occult brain metastasis during treatment, thus exaggerating the benefits of PCI.

At present, gadolinium-enhanced MRI is used for the diagnosis of brain metastases from SCLC, which is a more sensitive diagnostic imaging technique than CT for identifying brain metastases (MRI vs. CT: 24% vs. 10%) (10). In the era of brain MRI, the value of PCI in the treatment of LS-SCLC deserves further study. In our study, brain MRI was performed at the time of diagnosis and pre-PCI to exclude brain metastases, and the results revealed that PCI had nothing to do with the improvement of OS (mOS: 35 vs. 28 months, *p* = 0.128) and PFS (mPFS: 15 vs. 10 months, *p* = 0.186). Similarly, a retrospective study analyzed 297 LS-SCLC patients who underwent baseline brain MRI and restaging brain MRI and/or CT to exclude brain metastases. Among them, 209 patients received PCI, and PCI did not improve the OS of LS-SCLC patients (4-year OS: 32.1% vs. 28.6%, *p* = 0.32) (7). It is worth mentioning that although PCI did not achieve survival benefits in this study, it may be associated with a decline in the risk of developing new brain metastasis, thereby improving the quality of life of LS-SCLC patients. Previous studies have also confirmed the above conclusions. In a study published in Japan, 224 patients with extensive-stage SCLC (ES-SCLC) who had any response to chemoradiotherapy and no brain metastases on MRI were randomly assigned (113 to PCI and 111 to observation) (11). All patients underwent brain MRI at 3, 6, 9, 12, 18, and 24 months to monitor the intracranial state. The research showed that the use of PCI could reduce the cumulative incidence of brain metastasis (*p* < 0.0001) but did not achieve survival benefits (mOS: 10.1 vs. 15.1 months, *p* = 0.094).

In addition, a number of studies have shown that patients will experience a deterioration across time of nervous system function, intellectual deficit, and memory after PCI (12, 13). Gondi et al. analyzed the impact of PCI on cognitive functioning, and they considered that PCI is associated with a decline in Hopkins Verbal Learning Test at 6 months (odds ratio 3.91, 95% CI: 1.69–9.08, *p* = 0.002) and self-reported cognitive functioning at 6 months (odds ratio 3.60, 95% CI: 2.34–6.37, *p* < 0.0001) (14). In a Japanese study, due to concerns about neurotoxicity, only 12 of the 139 LS-SCLC patients received PCI treatment (15). In order to solve the problems mentioned above, PCI with the protection of the hippocampal neural stem-cell compartment has been recommended to preserve neurocognition function (16, 17). Recently, a phase III trial revealed that protection of the hippocampus during PCI could better preserve neurocognition function in patients with SCLC (18).

With the advancement of contemporary improvements in imaging and salvage techniques for brain metastases, the benefits of SRI have been increasing in SCLC. Ozawa et al. retrospectively studied the impact of MRI monitoring and SRI on the value of PCI in LS-SCLC patients. Patients with brain metastases during treatment were given priority to SRI treatment. The results showed no significant differences in OS (mOS: 25.0 vs. 34.0 months, *p* = 0.256) and the cumulative incidence of brain metastasis (*p* = 0.313) between the PCI and non-PCI groups (6). The JLGK0901 study reported that the SRI could effectively control up to ten brain metastases in cancer patients, including SCLC (19). Besides, the patient’s Mini-Mental State Examination score and radiation-related complications were under control after SRI treatment (20). Growing evidence from multiple retrospective studies has reported that SRI is an effective salvage treatment for SCLC patients who developed brain metastases after PCI or whole-brain radiotherapy (WBRT) (21, 22). For these reasons, the benefit of PCI may be attenuated for patients with MRI monitoring and SRI available.

In the present study, 48 patients (64.0%) and 45 (60.0%) experienced extracranial progression in the PCI and non-PCI groups, respectively. The CONVERT study showed that 449 (82%) LS-SCLC patients received PCI after completing chemoradiotherapy, of which 173 (39%) had extracranial progression (23, 24). Patients still have a high risk of extracranial progression after systemic treatment, and the occurrence of extracranial progression may affect the therapeutic value of PCI. Therefore, improvements in extracranial control may help patients further benefit from PCI. The application of immunotherapy can significantly improve the patient’s systemic disease control rate, which may help PCI to exert its therapeutic benefits. The IMpower133 and CASPIAN study showed that combining immunotherapy and chemotherapy can significantly prolong the PFS and OS of ES-SCLC patients, becoming a category 1 recommendation for ES-SCLC patients (25, 26). Similarly, for LS-SCLC, combining immunotherapy and chemoradiotherapy was well tolerated and yielded favorable outcomes (27). A study enrolled 40 patients with LS-SCLC; the researchers revealed that combining immunotherapy and chemoradiotherapy is beneficial for LS-SCLC patients, with the mOS and mPFS of 39.5 months (95% CI: 8.0–71.0) and 19.7 months (95% CI: 8.8–30.5), respectively. Therefore, in the era of immunotherapy, it is necessary to reassess the therapeutic value of PCI in SCLC patients.

It should be pointed out that the present study has limitations. First, this is a single-center retrospective study with patient heterogeneity and selection bias, although we attempted to reduce the influence of potential confounders through propensity score matching. Second, due to the limitations of the institutional database, we were unable to collect detailed information on patients’ cognitive function after PCI. Moreover, we did not further analyze the data regarding the specific salvage therapy strategies after brain metastases, which may affect survival. Further clinical trials may be warranted to confirm our results and determine the most suitable patients for PCI in the era of brain MRI.

## Conclusions

This retrospective study showed that when brain MRI was performed at the time of diagnosis and pre-PCI to exclude brain metastases, PCI could reduce the cumulative rate of brain metastases, but it did not achieve survival benefits for LS-SCLC patients. Therefore, in the contemporary era of brain MRI, prospective studies are warranted to confirm the value of PCI.

## Data Availability Statement

The raw data supporting the conclusions of this article will be made available by the authors, without undue reservation.

## Ethics Statement

This study was a retrospective study based on data obtained for clinical purposes. We have consulted extensively with the Ethics Committee of the Cancer Hospital of China Medical University, and they believe that our study does not require ethical approval. The Ethics Committee of Cancer Hospital of China Medical University gives up the ethical approval.

## Author Contributions

CQ and HY designed the research. CQ and HL performed the data acquisition. CQ and WL performed the statistical analysis. CQ, LZ, XS, and FW drafted the manuscript. All authors reviewed and approved the manuscript.

## Conflict of Interest

The authors declare that the research was conducted in the absence of any commercial or financial relationships that could be construed as a potential conflict of interest.

## Publisher’s Note

All claims expressed in this article are solely those of the authors and do not necessarily represent those of their affiliated organizations, or those of the publisher, the editors and the reviewers. Any product that may be evaluated in this article, or claim that may be made by its manufacturer, is not guaranteed or endorsed by the publisher.
